# Supplementary material for: Growth-rate dependency of ribosome abundance and translation elongation rate in Corynebacterium glutamicum differs from that in Escherichia coli
Source: Nat Commun. 2023 Sep 12;14:5611. doi: 10.1038/s41467-023-41176-y (PMC10497606; doi:10.1038/s41467-023-41176-y)
Supplement: Supplementary file 1 — Supplementary Information [file 41467_2023_41176_MOESM1_ESM.pdf]

Supplementary information

Growth-rate dependency of ribosome abundance and translation elongation rate in *Corynebacterium glutamicum* differs from that in *Escherichia coli*

Susana Matamouros, Thomas Gensch, Martin Cerff, Christian C. Sachs, Iman Abdollahzadeh, Johnny Hendriks, Lucas Horst, Niklas Tenhaef, Julia Tenhaef, Stephan Noack, Michaela Graf, Ralf Takors, Katharina Nöh and Michael Bott

Table of Contents

Supplementary Notes 3

Supplementary Note 1. SMLM ribosome counting with *SurEmCo* 3

Supplementary Note 2. Estimation of the number of ribosomes per cell from the total RNA. 6

Supplementary Note 3. Determination of the translation elongation rate via a fluorescent assay 7

Supplementary Note 4. Determination of the active ribosome fraction 9

Supplementary Note 5. Modelling the Rb/μ correlation 10

A. Preparing experimental data for modelling 10

B. Model overview and basic assumptions 11

C. Model calibration procedure 13

D. Discussion of the ribosome production rates in *C. glutamicum* 14

Supplementary Note 6. Comparison of modelling results for *C. glutamicum* (30 °C) and *E. coli* (30 °C and 37 °C) 16

Supplementary Figures 20

Supplementary Figure 1. uS2-PAmCherry and bL19-EYFP protein fusions are produced in strain SM34. 20

Supplementary Figure 2. Ribosome counting with the *SurEmCo* software. 22

Supplementary Figure 3. Ribosome number and cell volume for cultivation conditions indicated at the bottom. 23

Supplementary Figure 4. Assay for determination of the translation elongation rate using the EYFP fluorescent protein as reporter. 24

Supplementary Figure 5. Scheme of the coarse-grained self-replicator model and its most important modules and functions. 26
Supplementary Figure 6. Result of model calibration for *C. glutamicum* and *E. coli*. 27
Supplementary Figure 7. Comparison of the Rb/μ correlation, inferred rates and active ribosome fraction observed in *C. glutamicum* and *E. coli* 28
Supplementary Figure 8. Model-inferred tRNA loading status, active and inactive ribosome fractions, and ppGpp concentrations in dependency of the growth rate for *C. glutamicum* and *E. coli*. 30
Supplementary Figure 9. Simulation of the upshift experiment – interrogation of the model. . 32
Supplementary references 34

Supplementary Notes

Supplementary Note 1. SMLM ribosome counting with *SurEmCo*

The super-resolution emitter counter software *SurEmCo* (**https://github.com/modsim/suremco**) is developed in Python 3, using the numpy [1], scipy [2], numexpr, pandas [3] and OpenCV [4] libraries for data IO and processing. *SurEmCo* features an easy-to-use GUI (graphical user interface) provided by Qt (using PySide) and the VisPy library for 3D visualizations. Starting point for the analysis is a transmission image along with emitter positions previously reconstructed using SNSMIL. Cell regions are identified within the image (Supplementary Fig. 2a): the image is background corrected (by division with a Gaussian blurred version of itself), inverted, rescaled, and subsequently binarized using a local thresholding algorithm [5]. Cell regions smaller than 0.42 μm^2 and larger than 42.25 μm^2 were discarded, yielding a separation of cells from background. Cell clusters and superposed cells were manually identified and excluded from the downstream analysis. Cell lengths and widths are approximated by fitting ellipses to the cell contour. For each detected cell, emitters are collected and per-cell emitter tracking is performed (Supplementary Fig. 2b and 2c). Out of the different tracking algorithms available in *SurEmCo*, for performance reasons all images were analysed using a custom tracker written in C++, which implements an accelerated search by KD-trees [6] using the nanoflann library (https://github.com/jlblancoc/nanoflann).

In our study, emitter tracking is eased because the samples are fixed. Positional drifts in the obtained emitter traces may therefore only originate from movement of the sample stage, imperfect immobilization of the cells on the cover glass, residual mobility of the ribosomal

proteins in the fixed cell, and/or imprecise position determination in the single molecule localization measurements.

In emitter tracking, two potential SMLM issues were tackled that typically arise in the determination of the number of single molecules per cell, namely (1) the presence of the same fluorophore in consecutive frames and (2) the blinking probability of the fluorophore used. During SMLM, the fluorescence of all single molecules was collected until they were permanently photobleached. Under the applied illumination conditions (561 nm light power) the time until permanent photobleaching is ~50-1000 ms, *i.e.* a single photoactivated PAmCherry molecule is detectable in several consecutive images. The *SurEmCo* tracking algorithm that generates single-molecule time traces, checks for every detected emitter, whether there is an emitter in the previous image at that position, and counted every non-interrupted trace as one ribosomal protein (Supplementary Fig. 2c and 2d emitters #1 and #2). The second important aspect considered is the blinking probability of the fluorophore. The fluorescence output of most fluorophores is not constant, but shows intensity fluctuations with prominent periods of emission (ON-periods) interrupted by periods of no emission (OFF-periods), although the excitation is kept unchanged. This behaviour cannot be observed in ensemble measurements since it is averaged out. However, in studies on single fluorophores, where the molecules were immobilized and/or observed over long periods of time it becomes apparent that it is a common behaviour (see for instance [7,8]). When counting molecules, blinking becomes a problem since it introduces uncertainty on whether a new emerging photoactivated PAmCherry molecule is the result of a first-time photoactivation event or a molecule that returned from an interim OFF-state to an ON-state. To minimize the influence of the latter on the final emitter numbers by falsely counting

returning events possibly multiple times, the spatio-temporal relationship of the emerging tracks is analysed. If an emitter is detected at the position of a terminated track within one image frame after the termination, the absence is regarded as a blinking event, meaning it is considered to be the same emitter and part of the former track (Supplementary Fig. 2d emitter #3). This way the vast majority of the PAmCherry blinking events are accounted for. The detected emitters are displayed in a tabulated form (Supplementary Fig. 2e) for further processing.

Supplementary Note 2. Estimation of the number of ribosomes per cell from the total RNA.

In exponentially growing cells it is estimated that ~86% of total RNA is rRNA [9-11] and therefore the number of ribosomes in a sample can be calculated by $N_R = 0.86 \cdot R/m_{rRNA}$, where N_R is the total ribosome number, R is the total RNA content (here in 1 mL culture of $OD_{600} = 1$), and m_{rRNA} is the mass of rRNA per ribosome. *C. glutamicum* contains six *rrn* operons, which are very similar in sequence [12,13]. To calculate the mass of rRNA of a single ribosome, the 5S, 16S, and 23S rRNA sequences from the *rrnA* operon were used. They encompass 4,770 nucleotides (3,112 nt from 23S rRNA, 1,537 nt from 16S rRNA, 121 nt from 5S rRNA, 1,097 As, 1,000 Gs, 1,585 Cs, 1,088 Us) with a total molecular mass of 1,523 kDa or 2.53×10^{-15} mg. Mean colony forming units (cfu) per OD_{600} per mL were determined by plate enumeration of serial dilutions for two biological replicates of *C. glutamicum* wt grown either in BHI+GLU medium or CGXII+GLU medium until mid-exponential phase. Values of 2.39×10^8 (BHI+GLU) and 3.56×10^8 (CGXII+GLU) cfu/OD_{600}/mL were obtained for BHI+GLU and CGXII+GLU, respectively, and used for the calculation of the ribosome numbers per cell. From experimentally derived ribosome numbers N_R the *rrn* transcription initiation rate v_{rrn} can be obtained by multiplication with the corresponding specific growth rate μ as $v_{rrn} = N_R \cdot \mu$ [9].

Supplementary Note 3. Determination of the translation elongation rate via a fluorescent assay

The *C. glutamicum* MB001(DE3) strain carrying the reporter plasmid pMKEx2-*eyfp* was used for all translation assays. The strain was pre-cultivated overnight in the same medium as used in the assay. A BioLector cultivation system was used for automated recording of backscatter as a measure of growth and of fluorescence as a measure of translation. At the mid-exponential growth phase, expression of *eyfp* was induced with 5.5 mM IPTG and translation stopped by addition of 0.9 mg/mL chloramphenicol at 20-60 s intervals using a robotics platform to ensure accurate and reproducible intervals between experiments (Supplementary Fig. 4a). After stopping translation, the sample plates were incubated overnight at 30 °C to allow for complete maturation of the fluorophore. The specific fluorescence was then calculated from the ratio of fluorescence and backscatter values for each time point measured (every 5 min). After subtraction of the specific fluorescence of the no-IPTG control, the mean specific fluorescence obtained after the fluorescent signal of the samples had reached a constant maximum was then plotted for each translation arrest time point. Finally, a linear regression was used to determine the intersection point on the *x*-axis (Supplementary Fig. 4b and 4c). For some conditions, the plasmid pMKEx2-*mCherry-linker-eyfp* was used encoding the larger reporter protein mCherry-L-EYFP, which allowed determination of the time for translation initiation. Calculation of the translation elongation rate took into account the time for translation initiation, determined according to Zhu *et al.* [14] (Supplementary Datasetset 3). The translation elongation rate was calculated as the ratio between the length of the EYFP protein (238 aa) or mCherry-L-EYFP (508

aa) and the time at which fluorescence first appears, from which the time for translation initiation was subtracted.

For determination of the translation elongation rate of *E. coli*, all assays were performed with JM109(DE3) carrying pMKEXs-*efyp* in a Tecan Infinite M1000 Pro plate reader and the translation initiation time was assumed to be 10 s as previously determined [15].

Supplementary Note 4. Determination of the active ribosome fraction

The active ribosome fraction was calculated according to Dai *et al.* [15],

$$f_{active} = \frac{N_{activeRb}}{N_{Rb}} = \mu \frac{\sigma\prime}{k_R(\frac{R}{P})}$$

where σ' is a dimensionless constant given by $m_{rRNA}/(0.86 \cdot m_{aa})$. For *C. glutamicum* the m_{rRNA} is 1523 kDa (Supplementary Note 2). The average molecular mass of an amino acid (m_{aa}) is 110 Da, thus for *C. glutamicum* $\sigma' \approx 1.61\text{x}10^4$. The active ribosome fraction was then calculated from the values for growth rate (μ) and R/P ratio listed in Supplementary Dataset 5 and the translation elongation rate (k_R) values from Supplementary Dataset 3. The difference in μ values between Supplementary Dataset 3 and 5 comes from the fact that due to the experimental setup the same cultures could not be used for both the R/P ratio measurements and the translation elongation assays. Therefore, the mean μ between the biological replicates for the two assays was determined and used in the calculation of the active ribosome fraction.

Supplementary Note 5. Modelling the Rb/μ correlation

Here we describe the preparation of experimental data prior to modelling and the essential assumptions of the self-replicator (SR) model. In addition, further details about model calibration procedure for both, *C. glutamicum* and *E. coli*, and results from simulations are provided.

A. Preparing experimental data for modelling

Calculation of ribosomal protein fractions Φ_R: Fractions of total ribosomal protein per total protein (w/w) (Φ_R) were derived from measurements of total RNA per total protein (w/w) determined under steady-state conditions according to the procedure described in [16]. In short, Φ_R for a specific growth rate was obtained by multiplying RNA/protein ratios by a factor ρ [extended ribosome (µg)/RNA (µg)] [16]. As described in Scott *et al.*, the extended ribosome encompasses all ribosomal proteins plus their affiliates, which include all initiation and elongation factors as well as tRNA synthases, etc. ρ is calculated as the product of the three ratios: a) rRNA per total RNA (~86% w w^{-1}), b) ribosomal protein per rRNA (~53% w w^{-1}), and c) affiliated ribosomal proteins per ribosomal protein (~167% w w^{-1}). For *E. coli*, the value of ρ was estimated to be ~0.76 [16]. Due to the lack of organism-specific data for *C. glutamicum*, the same value was also used in this study.

Unifying specific growth rates μ: Where needed, specific growth rates μ (h^{-1}) were calculated from doubling times (min) by means of $\mu = \ln(2)/(\textit{doubling time}/60)$ or from doubling rates (doublings h^{-1}) by means of $\mu = \textit{doubling rate}\cdot\ln(2)$.

B. Model overview and basic assumptions

A schematic of the most important model components and variables is shown in Supplementary Fig. 5. For the full set of model equations, the reader is referred to the Source Code and the original study [17]. A listing of model parameters is found in Supplementary Dataset 6. Here, only the most relevant modelling assumptions are given:

- The specific growth rate μ (h^{-1}) is defined as the volumetric translation elongation rate v_R ($\mu mol_{aa}\ L_{cell}^{-1}\ s^{-1}$) per total amino acid concentration (total proteome) p ($\mu M_{aa,tot}$) of the cell (Eq. 1). Cell volume V_{cell} (L_{cell}^{-1}) is assumed constant in the model (Supplementary Dataset 6). v_R is determined by the maximum specific translation rate $k_{R,max}$, which is an independent parameter, and concentrations of ribosomal proteins r (µM), amino-acylated tRNA t_{aa} (µM) and free tRNA t_f (µM). The specific translation elongation rate k_R (aa ribosome^{-1} s^{-1}) was calculated by dividing v_R by r at a given nutrient condition. While v_R, k_R, t_{aa}, t_f, and r are model variables, the total proteome p is assumed to be constant and k_R a free parameter.
- The availability of the amino acids required for translation is reflected by the maximal specific rate of amino acid synthesis, referred to as nutrient quality parameter k_n (s^{-1}), which is (due to coarse-graining) an aggregated parameter that simultaneously captures the catalytic efficiency, extracellular nutrient quality, and nutrient concentrations.
- Ribosomal proteins are produced at a rate v_{rrn} (ribosomes s^{-1}), which is equivalent to (*rrn* transcription initiations s^{-1} $cell^{-1}$), that is derived from the maximum ribosome production rate $v_{rrn,max}$ and the ppGpp concentration ppGpp (µM) (Eq. 2). Herein, $v_{rrn,max}$ is an independent parameter.

- The fraction of ribosomal proteins Φ_R is determined by the concentration of all amino acids within the ribosomal proteins, calculated as $r \cdot N_{aa,r}$ (µM) per total proteome p (Eq. 3), where $N_{aa,r}$ is the number of amino acids per extended ribosome (12,307).
- Three different ribosome species are considered (Eq. 4):
  (1) Ribosomes that form complexes with amino acid-charged tRNA (r_{taa}).
  (2) Ribosomes that form complexes with uncharged tRNA (r_{tf}).
  (3) Free ribosomes that are not associated with any tRNA molecule (r_f).

  While the first ribosome species is considered to actively translating, the latter two species do not contribute to the translation elongation rate. Consequently, it is straightforward to calculate the fraction of actively translating ribosomes (f_{active}) (Eq. 5).

$$\mu = \frac{v_R}{p} = \frac{k_{R,max} \cdot r}{p} \cdot \left(1 + \sum_i \frac{1}{20}\left(\frac{\kappa_{taa}}{t_{aa_i}} + \frac{t_{f_i}}{t_{aa_i}} \cdot \frac{\kappa_{taa}}{\kappa_{tf}}\right)\right)^{-1} \qquad \text{(Eq. 1)}$$

$$v_{rrn} = v_{rrn,max} \cdot \frac{RNAP_F}{K_{M,rrn} + RNAP_F} \cdot \frac{1}{1 + \frac{ppGpp}{k_{i,ppGpp}}} \qquad \text{(Eq. 2)}$$

$$\Phi_R = (r/p) \cdot N_{aa,r} \qquad \text{(Eq. 3)}$$

$$r = r_{taa} + r_{tf} + r_f \qquad \text{(Eq. 4)}$$

$$f_{active} = \frac{r_{taa}}{r_{taa} + r_{tf} + r_f} \qquad \text{(Eq. 5)}$$

C. Model calibration procedure

The model calibration procedure was performed for *C. glutamicum*, for *E. coli* grown at 30 °C, and for *E. coli* grown at 37 °C, independently, using appropriate data sets of µ and R/P ratio. Data for *C. glutamicum* and some of the *E. coli* 30 °C were produced in this work (Supplementary Datasets 2, 4 and 9). Additional data for *E. coli* were taken from the literature, specifically from [18] for 30 °C and from [9,19] for 37 °C. This amounted to a total of 20 (30 °C) and 33 (37 °C) data points. In both cases, measurement errors of 10-15% were assumed according to values reported in [16]. The three data sets can be found in Supplementary Dataset 2 and in the Source Code.

Appropriate nutrient quality values k_n for each of the available growth conditions were found by means of the SR model (Supplementary Note 5B). Here, k_n is the effective maximal rate of amino acid synthesis per unit of metabolic enzyme that summarizes the catalytic efficiency of the overall cellular metabolism, the extracellular nutrient quality, nutrient concentration and related factors. Measurements for μ and Φ_R and the corresponding k_n values are listed in Supplementary Dataset 8. Upper and lower values for k_n were taken from [17]. Then, the key parameters, namely the maximum translation rate $k_{R,max}$ and the maximum ribosome production rate $v_{rrn,max}$, were estimated with the coarse-grained model from these sets of (μ, Φ_R, k_n)-triplets as described in the Methods. Measured and simulated data agreed very well for all strains and conditions (Fig. 4a). The sum of squared residuals (SSR) were ~22, ~49, and ~33 for *C. glutamicum*, *E. coli* 30 °C and *E. coli* 37 °C, respectively. These values were below the acceptable chi-squared test statistic of ~29, ~56 and ~84 at 95% significance level, respectively. Best parameter estimates for $k_{R,max}$ and $v_{rrn,max}$ including their associated standard deviations are found in Supplementary

Dataset 7 for *C. glutamicum* and *E. coli*. The resulting model fits are shown in Supplementary Fig. 6.

D. Discussion of the ribosome production rates in *C. glutamicum*

In the model v_{rrn} is determined from $v_{rrn,max}$ (Supplementary Dataset 7) and other parameters such as the parameter for free RNA polymerase concentration ($RNAP_f$) (Supplementary Dataset 6) by means of a pseudo kinetic (see (Eq. 2) in Supplementary Note 5B). Therefore, we only discuss how $v_{rrn,max}$ is qualitatively interrelated with the ribosome production rate v_{rrn}. A good match was found between the model-inferred ribosome production rate v_{rrn} and the rates independently approximated from the SMLM counted ribosome numbers (Supplementary Fig. 7c, Supplementary Note 2). Model-based inference shows that the ribosome production rate is ~3 newly extended ribosomes (*rrn* transcription initiations) per second and cell at μ = 0.4 h^{-1} (Supplementary Fig. 7c). At the predicted maximal growth rate of $\widehat{\mu}_{max}$ = 0.94 h^{-1}, enabled by using a hypothetical "super-rich" medium, an ribosome production rate of ~11 *rrn* transcription initiations s^{-1} $cell^{-1}$ was estimated. To summarize, values of $v_{rrn,max}$ (Supplementary Dataset 7) exceed those of the corresponding rates v_{rrn} (Supplementary Fig. 7c, dashed lines) by 1-2 orders of magnitude, which we attribute to the pseudo kinetics and their parameters, except v_{rrn} as explained before.

To unravel what ultimately limits ribosome production to achieve even higher growth rates > μ_{max}, it should be noted, that the concentration of uncharged tRNA (Supplementary Fig. 8b) and, therewith, the concentration of non-translating ribosome complexes drops towards zero

(Supplementary Fig. 8d). Modelling results reveal that, at higher growth rates, the insignificant number of the remaining non-translating ribosome complexes is connected to a very low ppGpp concentration (Supplementary Fig. 8e) limiting the further increase in ribosome production and therewith settling the maximum growth rate.

Supplementary Note 6. Comparison of modelling results for *C. glutamicum* (30 °C) and *E. coli* (30 °C and 37 °C)

Comparison between modelling results for *C. glutamicum* grown at 30 °C and *E. coli* grown at 30 °C and 37 °C revealed (after appropriate model calibration with literature data, see Methods, Supplementary Note 5C, and Supplementary Dataset 6) that *C. glutamicum* and *E. coli* at 30 °C require similar ribosome abundance to support comparable growth rates. These values are considerably higher compared to *E. coli* at 37 °C for growth rates μ = 0.4-1.2 h^{-1} (Supplementary Fig. 7a). For *E. coli*, the inferred maximum translation elongation rates were $k_{R,max}$ = 8.4 ± 0.4 aa $ribosome^{-1}$ s^{-1} at 30 °C and 17.7 ± 0.6 aa $ribosome^{-1}$ s^{-1} at 37 °C (Supplementary Dataset 7, Supplementary Fig. 7b), which is in line with previous experimental results [15,20]. *rrn* transcription initiation rates for *E. coli* were inferred to be v_{rrn} = 1.9 ± 0.7 – 19.0 ± 7.4 *rrn* transcription initiations s^{-1} $cell^{-1}$ at 30 °C and 2.4 ± 0.3 – 20.0 ± 2.5 *rrn* transcription initiations s^{-1} $cell^{-1}$ at 37 °C. While values of $k_{R,max}$ were comparable for *C. glutamicum* and *E. coli* at 30 °C, they only exhibited ~50% of the value obtained for *E. coli* grown at 37 °C. Similarly, at μ = 0.9 h^{-1} *C. glutamicum* and *E. coli* at 30 °C exhibit ~2-fold increased ribosome production rates compared to *E. coli* at 37 °C, as due to the lower translation rates at 30 °C a higher number of ribosomes is required to achieve the same growth rate (Supplementary Fig. 7c and Supplementary Note 5D).

The order of magnitude of model-derived ribosome production rates was experimentally verified for *E. coli*, where we assumed that 55,000 ribosomes at μ = 0.77 h^{-1} (30 °C) [21] and 72,000 ribosomes at μ = 1.72 h^{-1} (37 °C) [9] yield v_{rrn} = 9.5 and 34.4 *rrn* transcription initiations s^{-1} $cell^{-1}$, respectively (Supplementary Note 2 and Supplementary Fig. 7c). In line with the findings for

C. glutamicum, all values of $v_{rrn,max}$ (Supplementary Dataset 7) exceed those of the corresponding rates v_{rrn} (Supplementary Fig. 7c, dashed lines) by 1-2 orders of magnitude. Compared to *C. glutamicum*, maximum ribosome production rates $v_{rrn,max}$ were found to be 3.2- and 3.7-fold increased for *E. coli* grown at 30 °C and 37 °C, respectively. Consistent with this, also the ribosome production rates v_{rrn} obtained at large k_n values (super-rich medium, Supplementary Dataset 8) were found to be 2.7 and 2.5 fold increased for *E. coli* grown at 30 °C and 37 °C, respectively, when compared to *C. glutamicum*.

By increasing the specific rate of amino acid production (k_n, Supplementary Dataset 6), maximum specific growth rates $\hat{\mu}_{max}$ = 0.94, 1.46, and 2.03 h^{-1} were predicted for *C. glutamicum*, *E. coli* at 30 °C, and *E. coli* at 37 °C, respectively (see Methods, Supplementary Dataset 8, Supplementary Fig. 7a-d). Notably, the predicted maximal growth rates for *E. coli* are close to the maximum growth rates μ = 1.26 h^{-1} (30 °C) and 2.3 h^{-1} (37 °C) that were previously experimentally observed [18,22]. For *C. glutamicum*, as limiting factor for ribosome production (Supplementary Note 5D), and therefore higher growth rates, the model points to the limited availability of tRNAs (Supplementary Fig. 8b). As growth rate increases, in *C. glutamicum* the concentration of uncharged tRNA (Supplementary Fig. 8b) and, therewith, the concentration of non-translating ribosome complexes drops towards zero more rapidly than in *E. coli* (Supplementary Fig. 8d), and consequently settles a lower maximum growth rate. Because *E. coli* is known to employ active mechanisms of ribosome inactivation at growth rates $\mu < 0.5$ h^{-1} to maintain a relatively high translation rate at 37 °C (Supplementary Fig. 7b, [15]), a direct comparison of how *E. coli* and *C. glutamicum* utilize ribosomes at low growth rates is not possible with our model. Therefore,

regarding *E. coli* grown at 30 °C and 37 °C, the model was only calibrated with experimental data in the range $\mu \geq 0.31$ h^{-1} and $\mu \geq 0.5$ h^{-1}, respectively.

Due to the limited applicability of the model for *E. coli* (30 °C), since the model cannot explain the experimental data for growth rates lower than 0.3-0.4 h^{-1} (*i.e.* SSR >> 56, which is the acceptable chi-squared test statistic), simulated upshift experiments could only be compared when the experimental pre-shift growth rates were μ = 0.29 and 0.31 h^{-1} for *C. glutamicum* and *E. coli* (30°C), respectively (Supplementary Fig. 9d). Thus, pre-shift growth rates were relatively close to the common saturation post-shift growth rate μ = 0.46 h^{-1}. This implies that fractions of active ribosomes were ~98% for both strains before the shift (Supplementary Fig. 9f), and no explicit mechanistic insight could be gained into growth rate recovery when active ribosome fractions of only 50-90% were experimentally observed in *E. coli* (30°C) at μ << 0.31 h^{-1} (Supplementary Fig. 7d).

Time courses of selected variables before and after the shift to glucose medium are shown in Supplementary Fig. 9. All simulations were qualitatively and quantitatively very similar for the two organisms: while the pre-shift growth rate and ribosomal fraction Φ_R were 7% and 29% higher in *E. coli* (30°C), consequently, the immediate post-shift growth rate (at ~t_0+10s) was 19% higher in *E. coli* (30°C) (Supplementary Fig. 9d). Due to addition of nutrients at the shift (increase of k_n), the level of charged tRNA (t_{aa}/t_f) increased 80-100 fold within seconds after the shift (data not shown) and, concertedly, the fraction of active ribosomes immediately increased to ~99% in both strains (Supplementary Fig. 9f). Thus, translation rates in both strains immediately increased by 13-24% (Supplementary Fig. 9d), and consequently also the growth rates (see above and Eq.

1). Here, the immediate post-shift translation rate of *C. glutamicum* was ~8% above that of *E. coli* (which matches the ratio of 1.1 of the estimated $k_{R,max}$, for *C. glutamicum* and *E. coli* at 30°C, respectively, see Supplementary Dataset 7). In a second step, ppGpp-regulated *de novo* ribosome synthesis was triggered: in the transient phase, *i.e.* some minutes after the shift, ribosomes were newly synthesized at peak rates of 42 and 14 *rrn* transcription initiation s^{-1} in *E. coli* and *C. glutamicum* (Supplementary Fig. 9e), while the ppGpp decreased towards zero (Supplementary Fig. 9g). At its peak, ribosome synthesis was approx. three times higher in *E. coli* (which matches the ratio of 3.2 of the estimated $v_{rrn,max}$ for *C. glutamicum* and *E. coli* at 30°C, respectively, see Supplementary Dataset 7).

Supplementary Figures

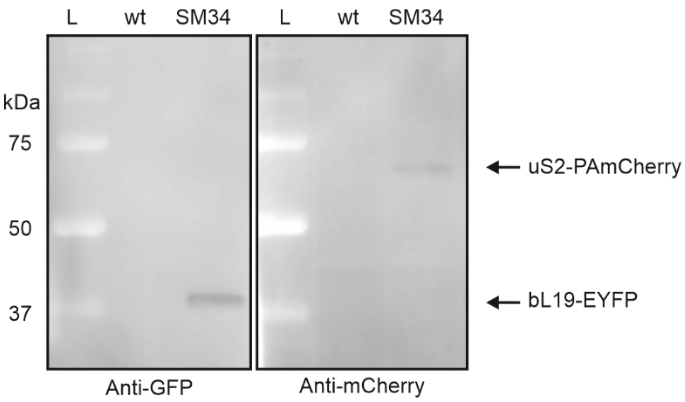

Supplementary Figure 1. uS2-PAmCherry and bL19-EYFP protein fusions are produced in strain SM34. Cell lysates of mid-exponential *C. glutamicum* wt and SM34 cultivated in BHI + glucose were separated by SDS-PAGE and analysed by western blot using the indicated antibodies. The molecular mass (kDa) of relevant proteins from the Dual Color Precision Plus Protein Prestained Standards (Bio-Rad) is shown on lanes L. Arrows indicate the position of the two fusion proteins in the SM34 extracts along with their predicted molecular mass. The experiments was performed twice with similar results.

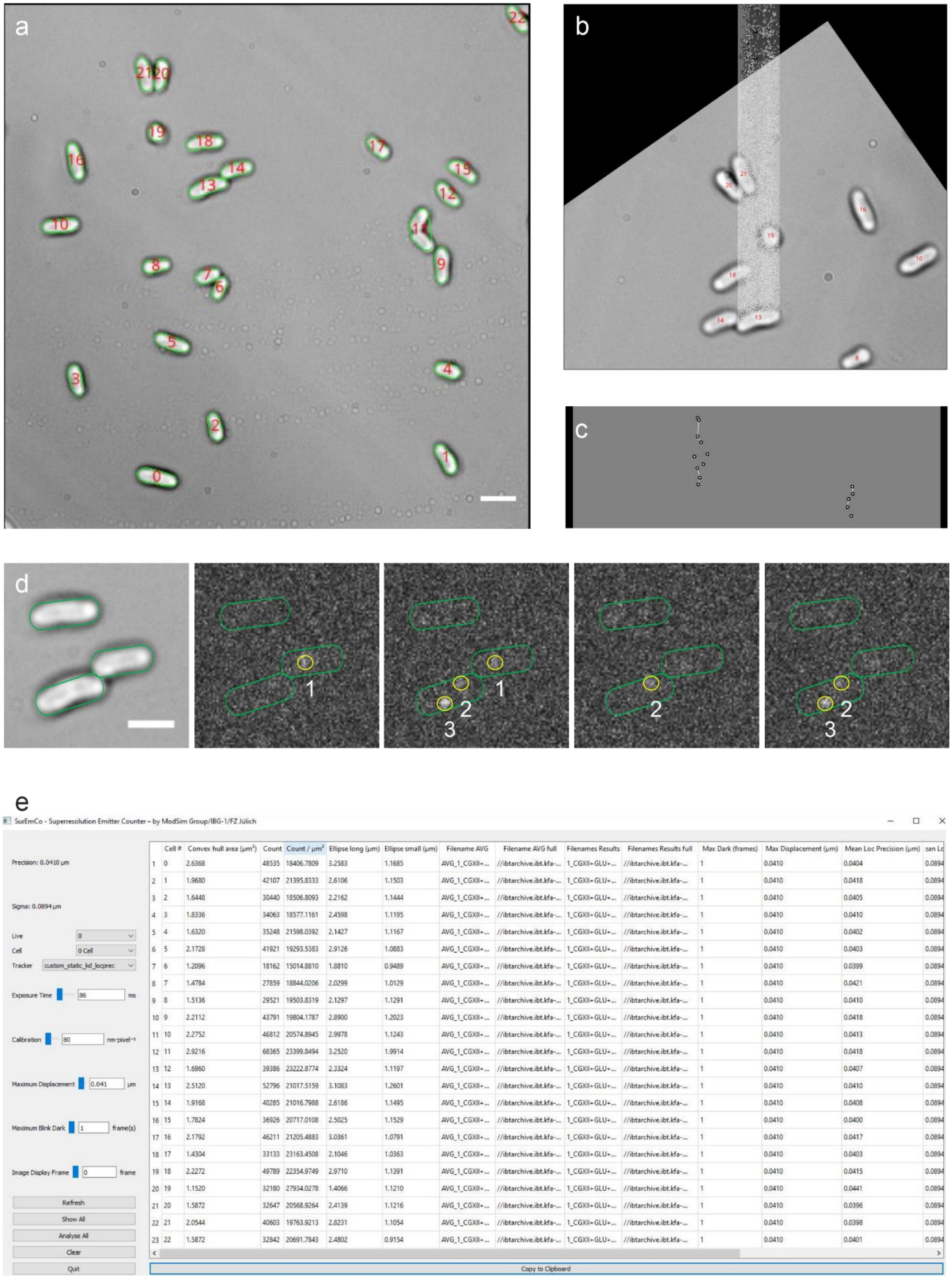
a
b
c
d
1
2
3
e
SurEmCo - Superresolution Emitter Counter – by ModSim Group/IBG-1/FZ Jülich
Precision: 0.0410 µm
Sigma: 0.0894 µm
Live 0
Cell 0 Cell
Tracker custom_static_kd_locprec
Exposure Time 86 ms
Calibration 80 nm·pixel⁻¹
Maximum Displacement 0.041 µm
Maximum Blink Dark 1 frame(s)
Image Display Frame 0 frame
Refresh
Show All
Analyse All
Clear
Quit
Cell # | Convex hull area (µm²) | Count | Count / µm² | Ellipse long (µm) | Ellipse small (µm) | Filename AVG | Filename AVG full | Filenames Results | Filenames Results full | Max Dark (frames) | Max Displacement (µm) | Mean Loc Precision (µm)
1 0 2.6368 48535 18406.7809 3.2583 1.1685 AVG_1_CGXII+... //ibtarchive.ibt.kfa-... 1_CGXII+GLU+... //ibtarchive.ibt.kfa-... 1 0.0410 0.0404 0.0894
2 1 1.9680 42107 21395.8333 2.6106 1.1503 AVG_1_CGXII+... //ibtarchive.ibt.kfa-... 1_CGXII+GLU+... //ibtarchive.ibt.kfa-... 1 0.0410 0.0418 0.0894
3 2 1.6448 30440 18506.8093 2.2162 1.1444 AVG_1_CGXII+... //ibtarchive.ibt.kfa-... 1_CGXII+GLU+... //ibtarchive.ibt.kfa-... 1 0.0410 0.0405 0.0894
4 3 1.8336 34063 18577.1161 2.4598 1.1195 AVG_1_CGXII+... //ibtarchive.ibt.kfa-... 1_CGXII+GLU+... //ibtarchive.ibt.kfa-... 1 0.0410 0.0410 0.0894
5 4 1.6320 35248 21598.0392 2.1427 1.1167 AVG_1_CGXII+... //ibtarchive.ibt.kfa-... 1_CGXII+GLU+... //ibtarchive.ibt.kfa-... 1 0.0410 0.0402 0.0894
6 5 2.1728 41921 19293.5383 2.9126 1.0883 AVG_1_CGXII+... //ibtarchive.ibt.kfa-... 1_CGXII+GLU+... //ibtarchive.ibt.kfa-... 1 0.0410 0.0403 0.0894
7 6 1.2096 18162 15014.8810 1.8810 0.9489 AVG_1_CGXII+... //ibtarchive.ibt.kfa-... 1_CGXII+GLU+... //ibtarchive.ibt.kfa-... 1 0.0410 0.0399 0.0894
8 7 1.4784 27859 18844.0206 2.0299 1.0129 AVG_1_CGXII+... //ibtarchive.ibt.kfa-... 1_CGXII+GLU+... //ibtarchive.ibt.kfa-... 1 0.0410 0.0421 0.0894
9 8 1.5136 29521 19503.8319 2.1297 1.1291 AVG_1_CGXII+... //ibtarchive.ibt.kfa-... 1_CGXII+GLU+... //ibtarchive.ibt.kfa-... 1 0.0410 0.0410 0.0894
10 9 2.2112 43791 19804.1787 2.8900 1.2023 AVG_1_CGXII+... //ibtarchive.ibt.kfa-... 1_CGXII+GLU+... //ibtarchive.ibt.kfa-... 1 0.0410 0.0418 0.0894
11 10 2.2752 46812 20574.8945 2.9978 1.1243 AVG_1_CGXII+... //ibtarchive.ibt.kfa-... 1_CGXII+GLU+... //ibtarchive.ibt.kfa-... 1 0.0410 0.0413 0.0894
12 11 2.9216 68365 23399.8494 3.2520 1.9914 AVG_1_CGXII+... //ibtarchive.ibt.kfa-... 1_CGXII+GLU+... //ibtarchive.ibt.kfa-... 1 0.0410 0.0418 0.0894
13 12 1.6960 39386 23222.8774 2.3324 1.1197 AVG_1_CGXII+... //ibtarchive.ibt.kfa-... 1_CGXII+GLU+... //ibtarchive.ibt.kfa-... 1 0.0410 0.0407 0.0894
14 13 2.5120 52796 21017.5159 3.1083 1.2601 AVG_1_CGXII+... //ibtarchive.ibt.kfa-... 1_CGXII+GLU+... //ibtarchive.ibt.kfa-... 1 0.0410 0.0410 0.0894
15 14 1.9168 40285 21016.7988 2.6186 1.1495 AVG_1_CGXII+... //ibtarchive.ibt.kfa-... 1_CGXII+GLU+... //ibtarchive.ibt.kfa-... 1 0.0410 0.0408 0.0894
16 15 1.7824 36926 20717.0108 2.5025 1.1529 AVG_1_CGXII+... //ibtarchive.ibt.kfa-... 1_CGXII+GLU+... //ibtarchive.ibt.kfa-... 1 0.0410 0.0400 0.0894
17 16 2.1792 46211 21205.4883 3.0361 1.0791 AVG_1_CGXII+... //ibtarchive.ibt.kfa-... 1_CGXII+GLU+... //ibtarchive.ibt.kfa-... 1 0.0410 0.0417 0.0894
18 17 1.4304 33133 23163.4508 2.1046 1.0363 AVG_1_CGXII+... //ibtarchive.ibt.kfa-... 1_CGXII+GLU+... //ibtarchive.ibt.kfa-... 1 0.0410 0.0403 0.0894
19 18 2.2272 49789 22354.9749 2.9710 1.1391 AVG_1_CGXII+... //ibtarchive.ibt.kfa-... 1_CGXII+GLU+... //ibtarchive.ibt.kfa-... 1 0.0410 0.0415 0.0894
20 19 1.1520 32180 27934.0278 1.4066 1.1210 AVG_1_CGXII+... //ibtarchive.ibt.kfa-... 1_CGXII+GLU+... //ibtarchive.ibt.kfa-... 1 0.0410 0.0441 0.0894
21 20 1.5872 32647 20568.9264 2.4139 1.1216 AVG_1_CGXII+... //ibtarchive.ibt.kfa-... 1_CGXII+GLU+... //ibtarchive.ibt.kfa-... 1 0.0410 0.0396 0.0894
22 21 2.0544 40603 19763.9213 2.8231 1.1054 AVG_1_CGXII+... //ibtarchive.ibt.kfa-... 1_CGXII+GLU+... //ibtarchive.ibt.kfa-... 1 0.0410 0.0398 0.0894
23 22 1.5872 32842 20691.7843 2.4802 0.9154 AVG_1_CGXII+... //ibtarchive.ibt.kfa-... 1_CGXII+GLU+... //ibtarchive.ibt.kfa-... 1 0.0410 0.0401 0.0894
Copy to Clipboard

Supplementary Figure 2. Ribosome counting with the *SurEmCo* software. *C. glutamicum* cells were cultivated in CGXII+GLU+CAA until mid-exponential phase, prepared for SMLM and imaged as described in the Methods. **a** – Cell regions are identified by the software. Each identified cell region is depicted on the original transmission image (green line delimitates the cell perimeter) and attributed an individual numerical identifier (red number). Scale bar – 3 µm. Similar results were obtained for all conditions tested. **b** – Emitters identified by the software within the cell boundaries are tracked in consecutive frames (3D projection). **c** – Emitter tracking (zoom into the 3D projection in **b**). Emitters (dots) that are located at the same position in subsequent image frames (within a precision threshold) are considered to belong to the same emitter. Emitter tracks are indicated by a white line. **d** – Examples of emitters counted as single events. #1 and #2 appear at the same position in consecutive frames. Likewise, since we chose a maximum blink dark value of one frame for all our analyses, emitter #3 was also counted as a single molecule. Scale bar – 2 µm. Similar observations were made for two biologically independent samples. **e** – Analysis parameters (left column) can be adjusted and results are displayed per cell on the right. *SurEmCo* is a Python tool that is available under the BSD license at https://github.com/modsim/suremco.

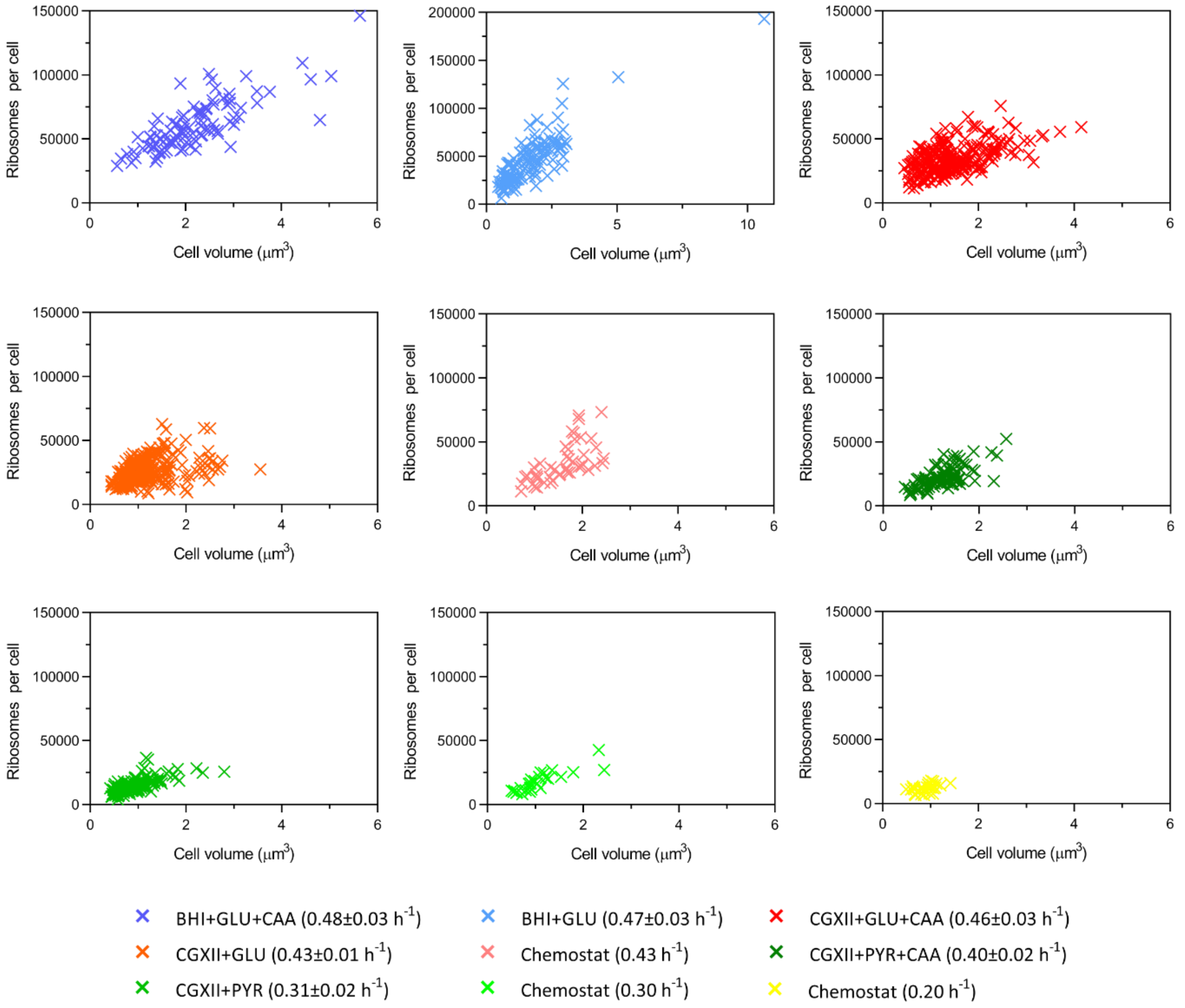

Supplementary Figure 3. Ribosome number and cell volume for cultivation conditions indicated at the bottom. PAmCherry counts as a proxy for the approximate number of ribosomes were determined by SMLM quantification for individual cells (each cross represents one cell) and are plotted in dependence of the cell volume. Cell volume was calculated from the width and length values of each cell determined by *SurEmCo* ($V = \pi \cdot W^2 \cdot (L - W/3)/4$) [23]. Colour code is the same as in Fig. 2c.

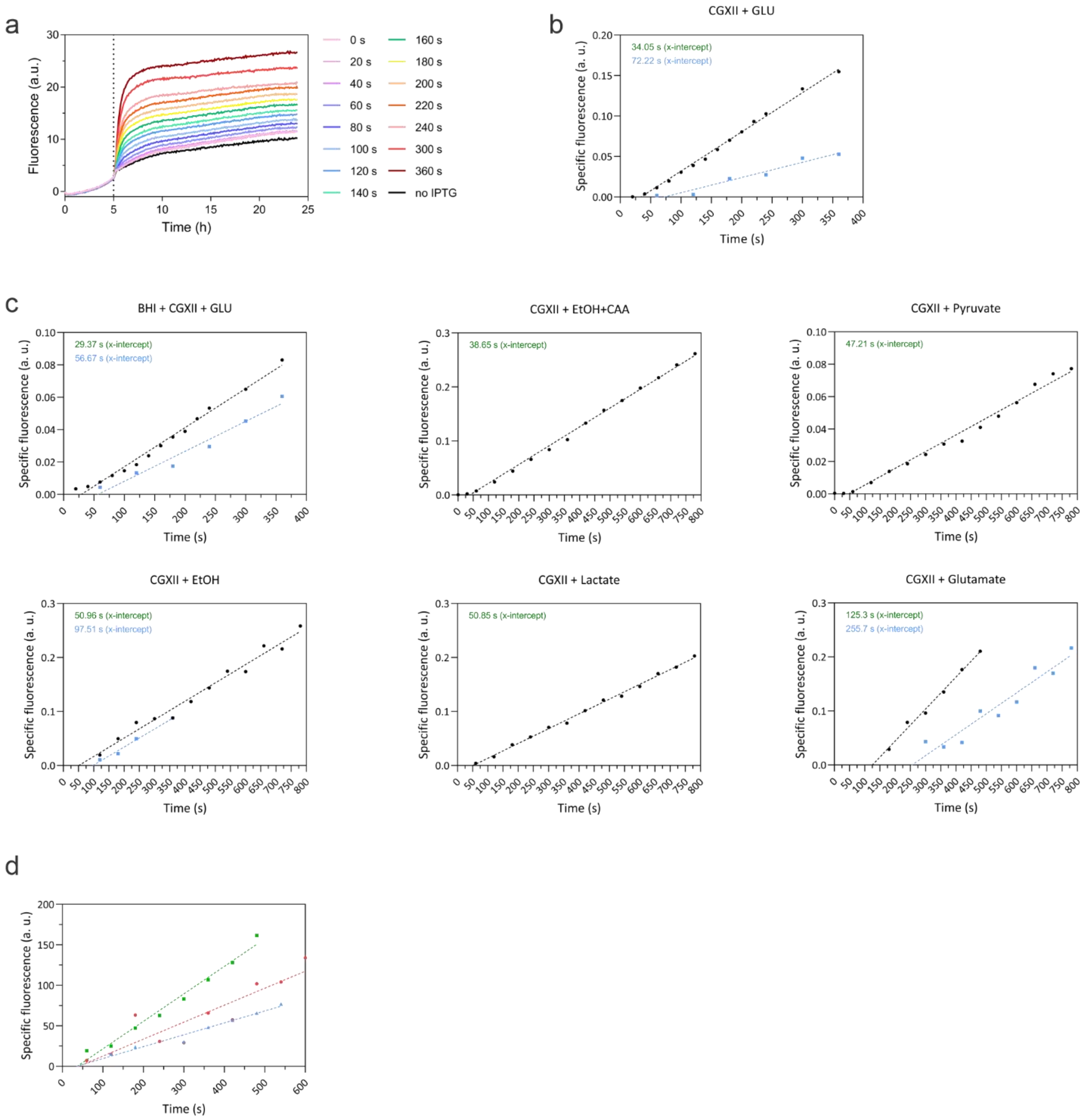

Supplementary Figure 4. Assay for determination of the translation elongation rate using the EYFP fluorescent protein as reporter. a – Strain *C. glutamicum* MB001(DE3) carrying pMKEx2-*eyfp* was grown in FlowerPlates^{TM} with CGXII+GLU medium using a robotics platform with an integrated Biolector cultivation system. At mid-exponential phase *eyfp* expression was induced by addition of 5.5 mM IPTG (vertical dashed line). Translation was stopped either immediately afterwards or 20-360 s later by chloramphenicol addition (0.9 mg/mL) and fluorescence was followed over time. **b** – The mean specific fluorescence of a period of constant maximum was plotted for each time point of chloramphenicol addition after normalization with the no-IPTG control.

A linear regression was used to determine the intersection point on the *x*-axis. The first EYFP (238 aa) fluorescence signal above the no-IPTG control was detected ~34 s after IPTG addition, not including the 7 s delay inherent to our robotic setup. The time for translation initiation (10.26 s) was subtracted, resulting in the first appearance of the EYFP signal after 30.79 s, giving a translation elongation rate of ~7.73 aa s^{-1} in this case. The data obtained for *C. glutamicum* carrying pMKEx2-*mCherry-linker-eyfp* encoding the mCherry-L-EYFP reporter composed of 508 aa is shown in blue, giving a translation elongation rate of 7.37 aa s^{-1}. **c** - Examples of translation elongation assays for all culture media tested. **d** - Examples of translation elongation assays for *E. coli* JM109(DE3) carrying pMKEx2-*eyfp* grown in MOPS+GLU (green), LB+GLU (red), and MOPS+ASP (blue).

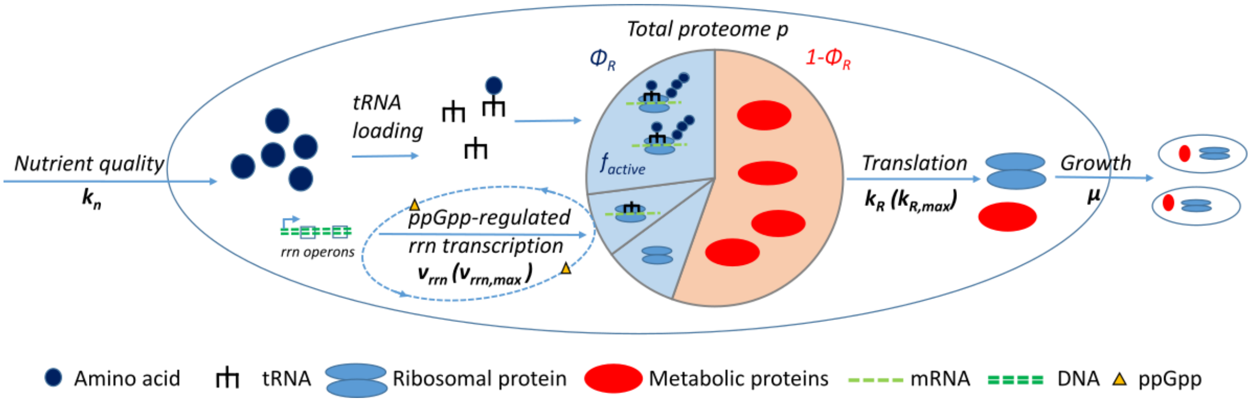

Supplementary Figure 5. Scheme of the coarse-grained self-replicator model and its most important modules and functions. In brief, amino acids are available in the cell as precursors for mRNA translation (k_R), to synthesize ribosomal and metabolic proteins, depending on the nutrient quality (k_n), *i.e.* the nutrient condition of the cell. Charged tRNAs-ribosome complexes constitute the fraction of actively translating ribosomes (f_{active}) within the fraction of ribosomal proteins (Φ_R) in the cell. Proteins synthesized are diluted into newly formed biomass expressed by the specific growth rate μ. The governing principle behind the SR model is rooted in the finite resource availability: too few ribosomes limit growth, while too many of ribosomes decrease the pool of available amino acids due to de novo ribosome synthesis that are, in turn, not available for growth. This reflects the cells' "balancing act" between amino acid flux and protein translation capacity and makes the SR model well suited to describe the phenomenological Rb/µ correlation. In the model, a ppGpp regulatory control mechanism senses suboptimal growth states. The control mechanism initiates compensatory *rrn* gene regulation to fine-tune ribosome production (v_{rrn}) and restore optimal growth. Calibrated with experimental data of *C. glutamicum* at hand, the model delivers quantitative insights into cellular key parameters of this species that were hitherto inaccessible or difficult to obtain experimentally (k_R, v_{rrn}). In addition, the calibrated model enables predictions. Inspired by illustrations from [15-17].

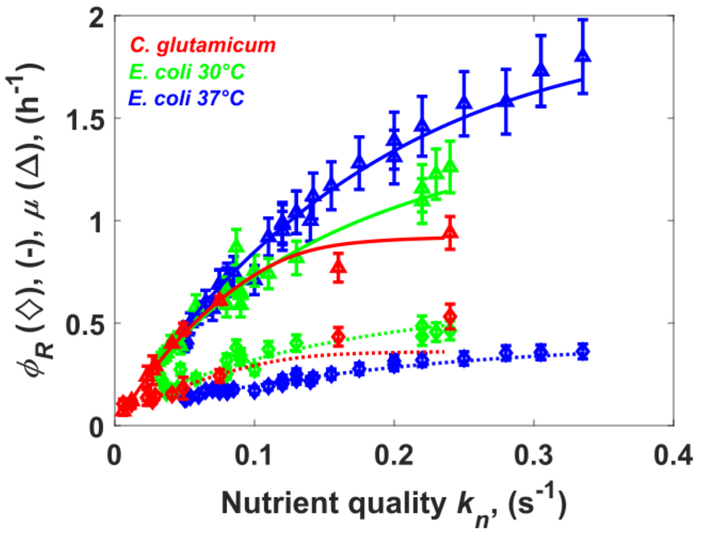

Supplementary Figure 6. Result of model calibration for *C. glutamicum* and *E. coli*. Model fits of specific growth rates μ (solid lines, 0.06 -1.8 h^{-1}) and ribosomal protein fractions Φ_R (dotted lines) over the range of k_n values for *C. glutamicum* (red, 0.006-0.240 s^{-1}), *E. coli* 30 °C (green, 0.02-0.24 s^{-1}) and *E. coli* 37 °C (blue, 0.05-0.34 s^{-1}). The mean and standard deviation is given for *C. glutamicum*: 0.94 h^{-1} (n=2); 0.77 h^{-1} (n=2); 0.61 h^{-1} (n=3), 0.47 h^{-1} (n=5), 0.40 h^{-1} (n=3); 0.29 h^{-1} (n=3); 0.27 h^{-1} (n=3); 0.24 h^{-1} (n=3); 0.12 h^{-1} (n=3); 0.07 h^{-1} (n=3), and for *E. coli* 30 °C: 0.87 h^{-1} (n=2), 0.58 h^{-1} (n=2) and 0.40 h^{-1} (n=2) where n is the number of biologically independent samples analysed (see also Supplementary Datasets 2 and 4). For the remaining *E. coli* data at 30 °C see [18,24] and for all data at 37 °C see [9,15,16,19]. Data used for model calibration is also contained in the Source Code. For the model calibration procedure see Material and Methods and Supplementary Note 5C.

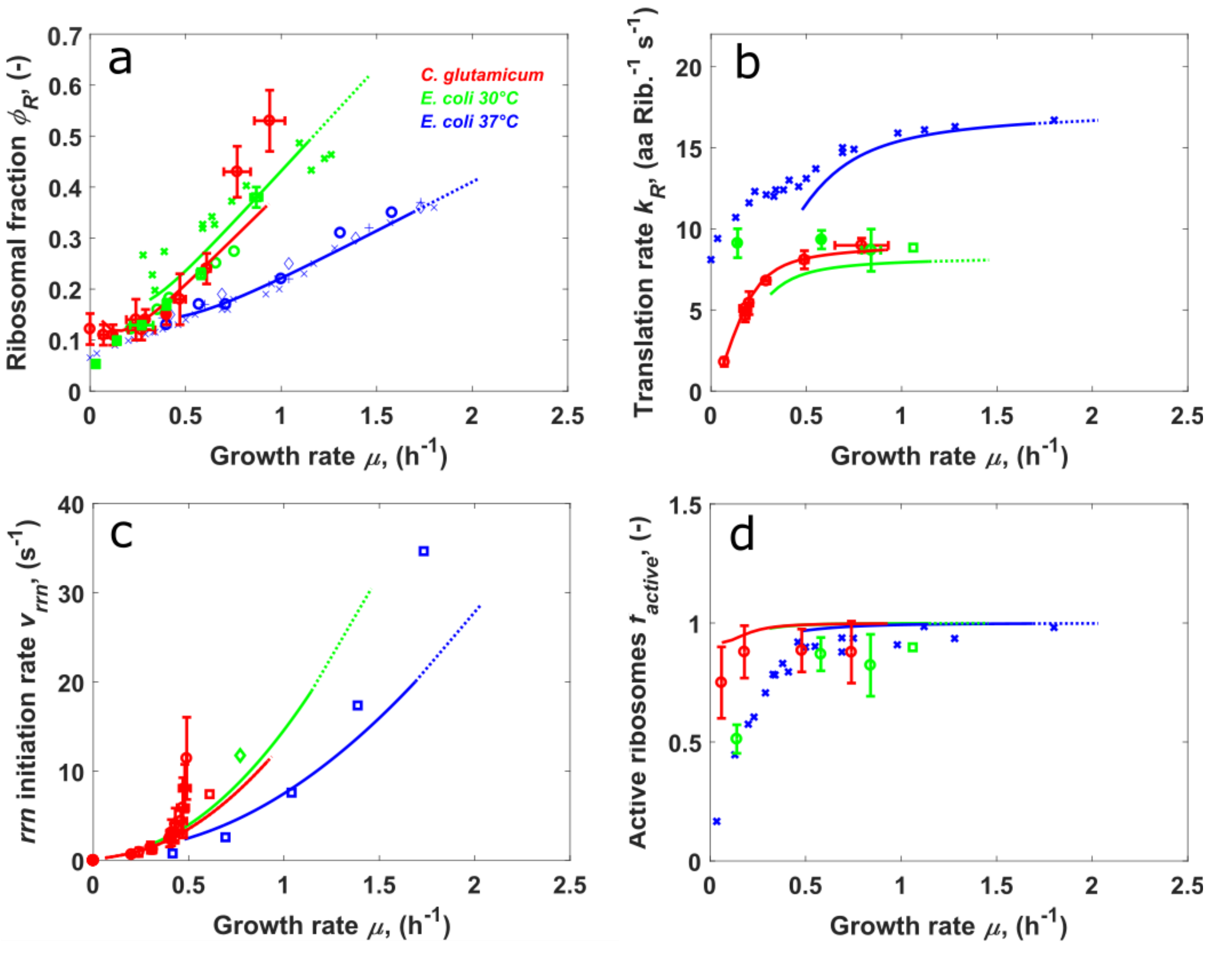

Supplementary Figure 7. Comparison of the Rb/μ correlation, inferred rates and active ribosome fraction observed in *C. glutamicum* and *E. coli*. a - Phenomenological observation of the Rb/μ correlation for *C. glutamicum* (this work), *E. coli* grown at 30 °C, and *E. coli* grown at 37 °C. Courses of ribosomal protein fractions Φ_R are shown at various growth rates. Experimental data is shown as symbols (see Supplementary Dataset 2 for *C. glutamicum*, Supplementary Dataset 4 for *E. coli* 30 °C and Source Code for *E. coli*). The number of biologically independent repeats is the same as given in Supplementary Figure 6. Additionally, the mean and standard deviation of Φ_R is also shown for *C. glutamicum* growing at: 0.0 h^{-1} (n=2), and for *E. coli* 30 °C growing at: 0.27 h^{-1} (n=2), 0.14 h^{-1} (n=2) and 0.03 h^{-1} (n=2) where n is the number of biologically independent samples analyzed. For the remaining *E. coli* data at 30 °C see [18,24] and for all data at 37 °C see [9,15,16,19]. *E. coli* experimental data was taken from the literature (**o**) [16], (**+**) [19], (**◊**) [9], (**x**) [15], (**o**) [24], (**x**) [18], except for (□), which was measured in the course of this work (see Supplementary Dataset 4). Lines represent respective simulated values using the calibrated model with estimated parameters (Supplementary Dataset 7): *C. glutamicum* (red), *E. coli* 30 °C (green) and *E. coli* 37 °C (blue), respectively. The range of the calibrated model is represented

by the solid lines. Dashed line extensions to the solid lines represent growth under "super-rich medium" represented by k_n increased by two orders of magnitude (Supplementary Dataset 8). **b** - Comparison of experimental (symbols) and of model-derived (solid lines) translation rates k_R. *E. coli* experimental data was retrieved from the literature: (□) [20], (**x**) [15]. Translation elongation rates of *C. glutamicum* and *E. coli* (□) determined in this work are listed in Supplementary Dataset 3 and 4, respectively. Depicted are mean values and standard deviation for the translation elongation rate for the following growth rates: 0.79 h^{-1} (n=3); 0.49 h^{-1} (n=2); 0.29 h^{-1} (n=2); 0.20 h^{-1} (n=3); 0.18 h^{-1} (n=2); 0.18 h^{-1} (n=3); 0.07 h^{-1} (n=2) for *C. glutamicum* (see also Supplementary Dataset 3) and 0.84 h^{-1} (n=2); 0.58 h^{-1} (n=2); 0.14 h^{-1} (n=4) for *E. coli* 30 °C (see also Supplementary Dataset 4) where n is the number of biologically independent repeats performed for each condition. Data for *E. coli* 37 °C can be taken from the Source Code. **c** - Comparison of experimental and model-derived ribosome production rates (*i.e. rrn* transcription initiation rates). *C. glutamicum* data was calculated from SMLM data (**o**) and from total RNA per cfu data (□) (see Supplementary Dataset 1 and Supplementary Note 2). Shown is the mean and standard deviation for n=3 biologically independent experiments for CGXII+PYR, CGXII+PYR+CAA, BHI+GLU and BHI+GLU+CAA or n=4 biologically independent experiments for CGXII+GLU and CGXII+GLU+CAA (see also Supplementary Dataset 1). *E. coli* experimental data (□) and (**◊**) was calculated from [9] and [21], respectively. *E. coli* data is contained within the Source Code. The rate of *rrn* transcription initiation v_{rrn} was calculated according to $v_{rrn} = N_R \cdot \mu$ [9] and was experimentally determined by measuring the number of ribosomes per cell N_R at the corresponding specific growth rate μ (s^{-1}). v_{rrn} is also termed ribosome production rate because it is assumed that each *rrn* transcription initiation triggers the production of one extended ribosome (Supplementary Note 5A). **d** - Comparison of experimental and model-derived fractions f_{active} of actively translating ribosomes. For *C. glutamicum* the mean and standard deviation is shown. Please refer to the Supplementary Datasets 4 and 5 for the number of biologically independent repeats performed for the calculation of the active ribosome fraction. All experimental and simulated data is also contained in the Source Code. For *E. coli* 37 °C experimental raw data (corresponding sets of μ, k_R, total RNA per total protein (w/w)) were taken from [15] and fractions of active ribosomes (**x**) calculated according to Supplementary Note 4 assuming $\sigma'_{E. coli(37°C)} = m_{RNA} / (m_{aa} \cdot 0.86) = 1.56 \cdot 10^4 \approx \sigma'_{E. coli(30°C)}$ [15]. For *E. coli* 30 °C (□), experimental raw data for k_R and μ were taken from [20], and the fraction of total RNA per total protein was recalculated from $\Phi_{R,sim}$ at μ = 1.06 h^{-1} (see **a**) for calculation of the active ribosome fraction.

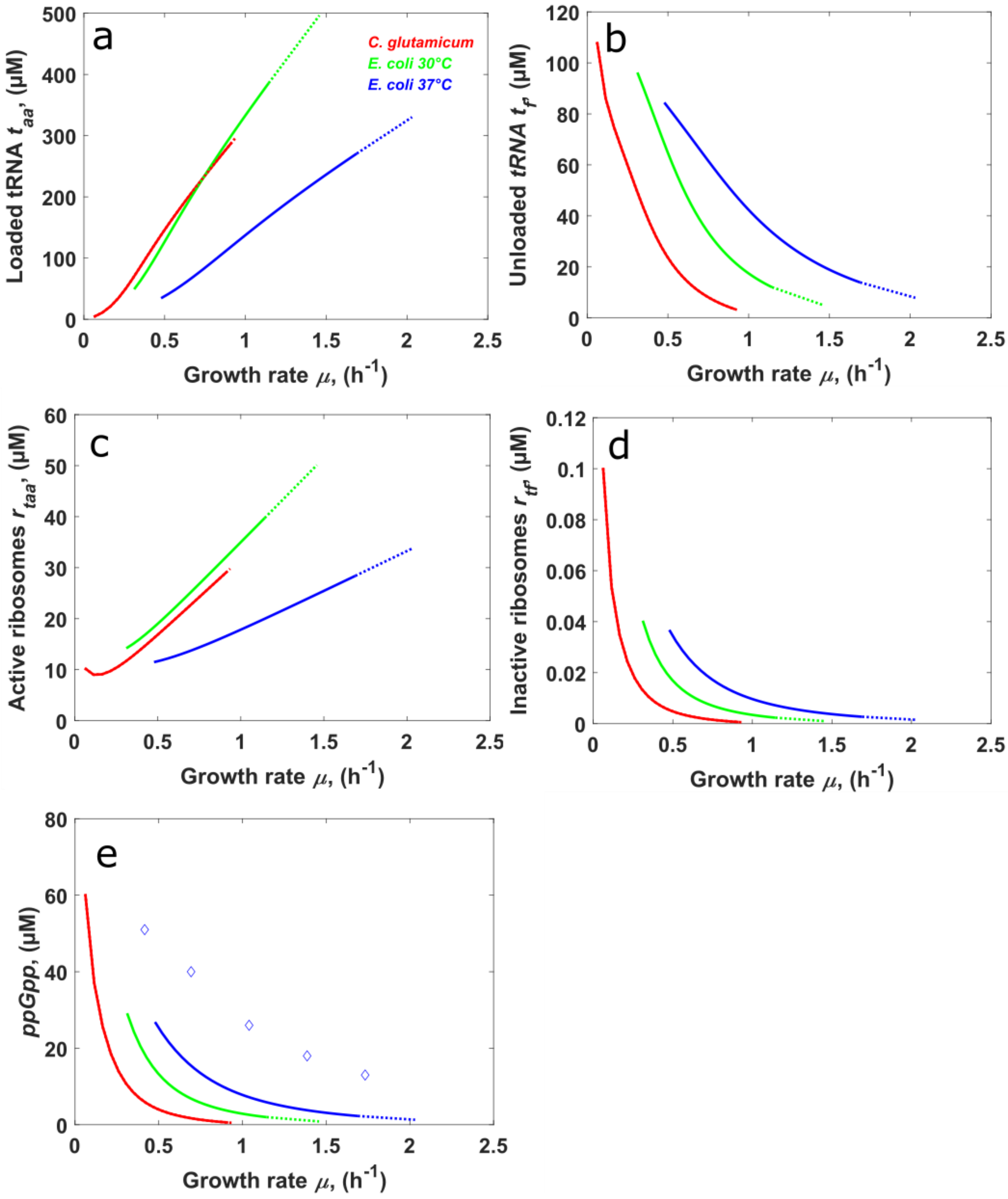

Supplementary Figure 8. Model-inferred tRNA loading status, active and inactive ribosome fractions, and ppGpp concentrations in dependency of the growth rate for *C. glutamicum* and *E. coli*. This figure summarizes for the indicated growth rates the concentrations of **a –** charged tRNA (t_{aa}), **b –**uncharged tRNA (t_f), **c –** complexes of actively translating ribosomes (r_{taa}), **d –** non-translating complexes due to bound uncharged tRNA (r_{tf}), **e –** ppGpp. Experimental data (◊) for *E. coli* was taken from [9].

Dashed lines represent model predictions for growth under “super-rich” medium, represented by k_n increased by two orders of magnitude (see Supplementary Dataset 8). All data can be found in the Source Code.

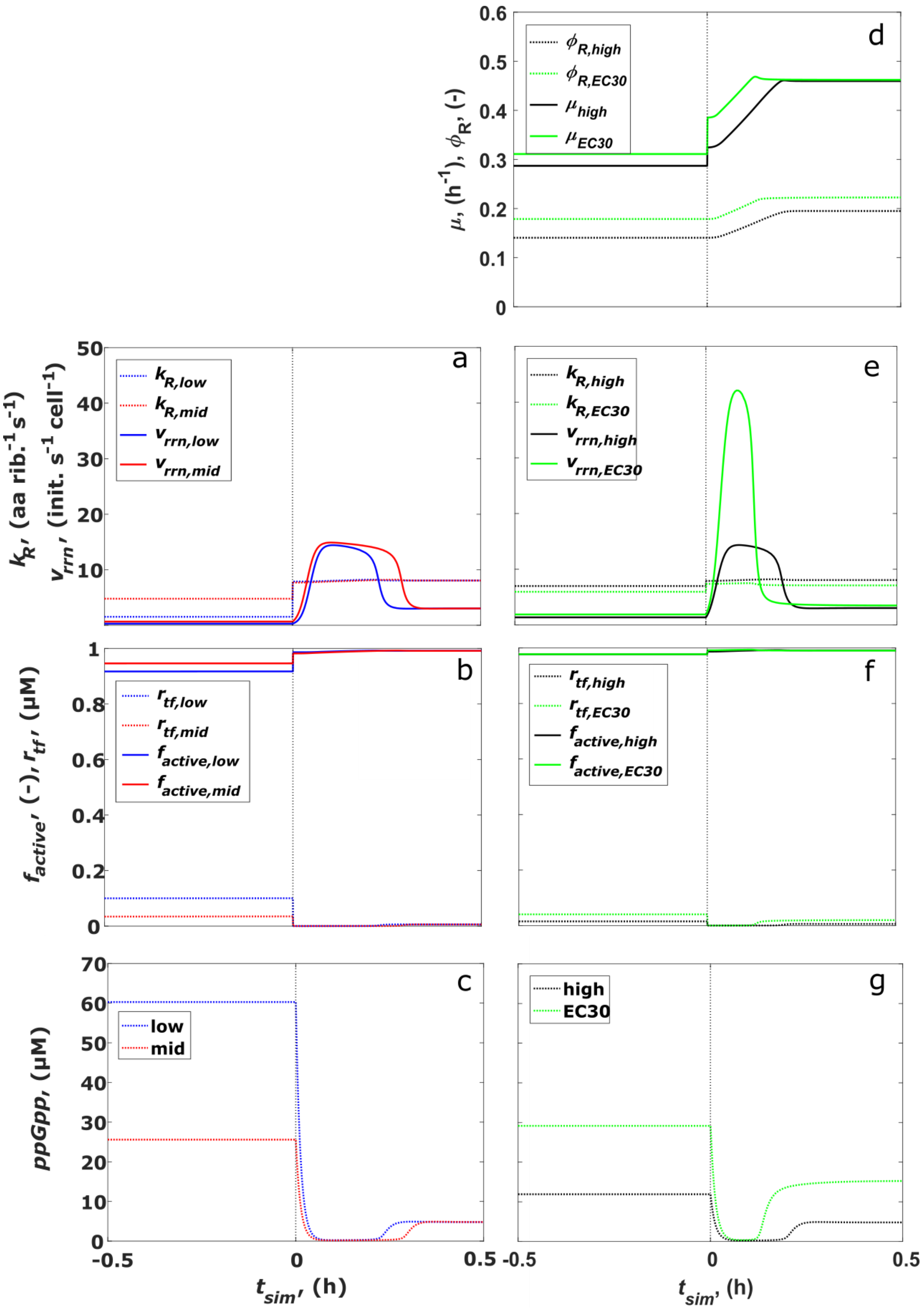

Supplementary Figure 9. Simulation of the upshift experiment – interrogation of the model. Comparison of inferred instantaneous translation elongation k_R as well as ribosome production rates v_{rrn} **(a, e),** instantaneous concentrations of inactive (uncharged tRNA-) ribosome complexes r_{tf} and active ribosome fractions f_{active} **(b, f)** and the ppGpp concentration **(c, g)** and **(d)** ribosome fractions Φ_R and growth rates μ (see also Fig. 5d). The nutrient shift (vertical dashed line at t=0) was initiated by an

increase of the nutrient quality parameter (see Methods): for *C. glutamicum* the upshift was simulated either from $k_{n,glutamate}$ = 0.006 s^{-1} (index "low", blue curves) or $k_{n,EtOH}$ = 0.016 s^{-1} (index "mid", red curves) or $k_{n,pyruvate}$ = 0.029 s^{-1} (index "high", black curves) to $k_{n,glucose}$ = 0.051 s^{-1} (Supplementary Dataset 9). For *E. coli* grown at 30°C (EC30) the upshift was simulated from k_n = 0.033 s^{-1} (index "EC30", green curves) to $k_{n,glucose}$ = 0.053 s^{-1} (Supplementary Dataset 4). For *E. coli*, similar k_n values were selected before and after the nutrient upshift, compared to *C. glutamicum* (black lines, "high" condition), for better comparability between the two strains. All data can be taken from the Supplementary Dataset File "Upshift Simulations".

Supplementary references

1 van der Walt, S., Colbert, S. C. & Varoquaux, G. The NumPy array: a structure for efficient numerical computation. *Comput Sci Eng* **13**, 22-30 (2011).

2 Virtanen, P. *et al.* SciPy 1.0: fundamental algorithms for scientific computing in Python. *Nat Methods* (2020).

3 McKinney, W. Data structures for statistical computing in Python. *Proc. of the 9th Python in Science Conf. (SciPy 2010)* (2010).

4 Bradski, G. The OpenCV library. *Dr. Dobb's Journal of Software Tools* **120**, 122-125 (2000).

5 Sauvola, J., Seppanen, T., Haapakoski, S. & Pietikainen, M. Adaptive document binarization. *Proc Int Conf Doc* **1**, 147-152 (1997).

6 Bentley, J. L. Multidimensional Binary Search Trees Used for Associative Searching. *Commun Acm* **18**, 509-517 (1975).

7 Gensch, T., Bohmer, M. & Aramendia, P. F. Single molecule blinking and photobleaching separated by wide-field fluorescence microscopy. *J Phys Chem A* **109**, 6652-6658 (2005).

8 Hofkens, J. *et al.* Triplet states as non-radiative traps in multichromophoric entities: single molecule spectroscopy of an artificial and natural antenna system. *Spectrochim Acta A Mol Biomol Spectrosc* **57**, 2093-2107 (2001).

9 Bremer, H. & Dennis, P. P. Modulation of chemical composition and other parameters of the cell at different exponential growth rates. *EcoSal Plus* **3** (2008).

10 Dennis, P. P. & Bremer, H. Differential rate of ribosomal protein synthesis in *Escherichia coli* B/r. *J Mol Biol* **84**, 407-422 (1974).

11 Neidhardt, F. C. & Magasanik, B. Studies on the role of ribonucleic acid in the growth of bacteria. *Biochim Biophys Acta* **42**, 99-116 (1960).

12 Martin, J. F., Barreiro, C., Gonzalez-Lavado, E. & Barriuso, M. Ribosomal RNA and ribosomal proteins in corynebacteria. *J Biotechnol* **104**, 41-53 (2003).

13 Tauch, A. *et al.* Strategy to sequence the genome of *Corynebacterium glutamicum* ATCC 13032: use of a cosmid and a bacterial artificial chromosome library. *J Biotechnol* **95**, 25-38 (2002).

14 Zhu, M., Dai, X. & Wang, Y. P. Real time determination of bacterial *in vivo* ribosome translation elongation speed based on LacZalpha complementation system. *Nucleic Acids Res* **44**, e155 (2016).

15 Dai, X. *et al.* Reduction of translating ribosomes enables *Escherichia coli* to maintain elongation rates during slow growth. *Nat Microbiol* **2**, 16231 (2016).

16 Scott, M., Gunderson, C. W., Mateescu, E. M., Zhang, Z. & Hwa, T. Interdependence of cell growth and gene expression: origins and consequences. *Science* **330**, 1099-1102 (2010).

17 Bosdriesz, E., Molenaar, D., Teusink, B. & Bruggeman, F. J. How fast-growing bacteria robustly tune their ribosome concentration to approximate growth-rate maximization. *FEBS J* **282**, 2029-2044 (2015).

18 Rosset, R., Julien, J. & Monier, R. Ribonucleic acid composition of bacteria as a function of growth rate. *J Mol Biol* **18**, 308-320 (1966).

19 Forchhammer, J. & Lindahl, L. Growth rate of polypeptide chains as a function of the cell growth rate in a mutant of *Escherichia coli* 15. *J Mol Biol* **55**, 563-568 (1971).

20 Farewell, A. & Neidhardt, F. C. Effect of temperature on in vivo protein synthetic capacity in *Escherichia coli*. *J Bacteriol* **180**, 4704-4710 (1998).

21 Bakshi, S., Siryaporn, A., Goulian, M. & Weisshaar, J. C. Superresolution imaging of ribosomes and RNA polymerase in live *Escherichia coli* cells. *Mol Microbiol* **85**, 21-38 (2012).

22 Schmidt, A. *et al.* The quantitative and condition-dependent *Escherichia coli* proteome. *Nat Biotechnol* **34**, 104-110 (2016).
23 Heldal, M., Norland, S. & Tumyr, O. X-ray microanalytic method for measurement of dry matter and elemental content of individual bacteria. *Appl Environ Microbiol* **50**, 1251-1257 (1985).
24 Rosset, R., Monier, R. & Julien, J. RNA composition of *Escherichia Coli* as function of growth rate. *Biochem Bioph Res Co* **15**, 329-333 (1964).
