# Supplementary material for: Growth-rate dependency of ribosome abundance and translation elongation rate in Corynebacterium glutamicum differs from that in Escherichia coli
Source: Nat Commun. 2023 Sep 12;14:5611. doi: 10.1038/s41467-023-41176-y (PMC10497606; doi:10.1038/s41467-023-41176-y)
Supplement: Supplementary file 3 — Description of Additional Supplementary Files [file 41467_2023_41176_MOESM3_ESM.pdf]

Description of Supplementary Datasets:

Supplementary Dataset 1: SMLM dataset for ribosome quantification in *C. glutamicum* cells grown at different growth rates.

Supplementary Dataset 2: RNA-protein (R/P) ratio of *C. glutamicum* at different growth rates.

Supplementary Dataset 3: Translation elongation rate (kR) and translation initiation time of *C. glutamicum* at different growth rates.

Supplementary Dataset 4: RNA-protein (R/P) ratio, translation elongation rate, and active ribosome fraction of *E. coli* growing at different growth rates at 30 °C.

Supplementary Dataset 5: Fraction of active ribosomes of *C. glutamicum* at different growth rates.

Supplementary Dataset 6: Model parameters and variables.

Supplementary Dataset 7: Estimated model parameters.

Supplementary Dataset 8: Prediction of maximum specific growth rate and ribosome protein fractions for high hypothetical nutrient qualities k_n.

Supplementary Dataset 9: Dataset for estimation of model parameters $k_{R,max}$ and $v_{rrn, max}$.

Supplementary Dataset 10: Summary of growth rate dataset of the upshift experiments conducted experimentally and *in silico*.
