# Supplementary material for: Growth-rate dependency of ribosome abundance and translation elongation rate in Corynebacterium glutamicum differs from that in Escherichia coli
Source: Nat Commun. 2023 Sep 12;14:5611. doi: 10.1038/s41467-023-41176-y (PMC10497606; doi:10.1038/s41467-023-41176-y)
Supplement: Supplementary file 5 — Reporting Summary [file 41467_2023_41176_MOESM5_ESM.pdf]

nature portfolio

Corresponding author(s): Susana Matamouros, Michael Bott

Last updated by author(s): August 17,2023

Reporting Summary

Nature Portfolio wishes to improve the reproducibility of the work that we publish. This form provides structure for consistency and transparency in reporting. For further information on Nature Portfolio policies, see our Editorial Policies and the Editorial Policy Checklist.

Statistics

For all statistical analyses, confirm that the following items are present in the figure legend, table legend, main text, or Methods section.

n/a	Confirmed	
☐	☒	The exact sample size (*n*) for each experimental group/condition, given as a discrete number and unit of measurement
☐	☒	A statement on whether measurements were taken from distinct samples or whether the same sample was measured repeatedly
☒	☐	The statistical test(s) used AND whether they are one- or two-sided<br>*Only common tests should be described solely by name; describe more complex techniques in the Methods section.*
☒	☐	A description of all covariates tested
☐	☒	A description of any assumptions or corrections, such as tests of normality and adjustment for multiple comparisons
☐	☒	A full description of the statistical parameters including central tendency (e.g. means) or other basic estimates (e.g. regression coefficient) AND variation (e.g. standard deviation) or associated estimates of uncertainty (e.g. confidence intervals)
☒	☐	For null hypothesis testing, the test statistic (e.g. *F*, *t*, *r*) with confidence intervals, effect sizes, degrees of freedom and *P* value noted<br>*Give P values as exact values whenever suitable.*
☒	☐	For Bayesian analysis, information on the choice of priors and Markov chain Monte Carlo settings
☒	☐	For hierarchical and complex designs, identification of the appropriate level for tests and full reporting of outcomes
☒	☐	Estimates of effect sizes (e.g. Cohen's *d*, Pearson's *r*), indicating how they were calculated

Our web collection on statistics for biologists contains articles on many of the points above.

Software and code

Policy information about availability of computer code

Data collection	Images were recorded with an EMCCD camera (Andor iXon DU897E-C00-#BV, Oxford instruments, Abingdon, UK) cooled to -75 °C using a resolution of 512 x 512 pixels. Data acquisition and storage was performed with the help of routines from the Andor Software Development Kit version 2.99.30007.0 (Oxford Instruments, UK)<br>Backscatter and fluorescence measurements were performed on a microcultivation system recorded using BioLection v.2.4.1.0 (m2p-labs GmbH)
Data analysis	Fiji (ImageJ) v2.0.0-rc-43/1.52n (open source)<br>GraphPad Prism 9.1.2 (GraphPad Software, Inc.)<br>SNSMIL v 1.01(open source)<br>SNSMIL rendering tool (open source)<br>Matlab v2016b (Mathworks)<br>The custom computer algorithm used in the analysis of SMLM data is deposited in Github (https://github.com/modsim/suremco)<br>Python v3.2.2

For manuscripts utilizing custom algorithms or software that are central to the research but not yet described in published literature, software must be made available to editors and reviewers. We strongly encourage code deposition in a community repository (e.g. GitHub). See the Nature Portfolio guidelines for submitting code & software for further information.

Data

Policy information about availability of data

All manuscripts must include a data availability statement. This statement should provide the following information, where applicable:

- Accession codes, unique identifiers, or web links for publicly available datasets
- A description of any restrictions on data availability
- For clinical datasets or third party data, please ensure that the statement adheres to our policy

Source Data file 1 contains the source data for figures 1a, 2b-d, 3a-c, 4a-c, 5a-d, Supplementary Data 3, 4, 5 and Supplementary Figures 1, 4a-d as well as an indication where to find the source data for Supplementary Figures 6-9.
Source Data file 2 contains all the source data for the SMLM used to generate figures 2b-d, 3 and Supplementary Data 1.

Research involving human participants, their data, or biological material

Policy information about studies with human participants or human data. See also policy information about sex, gender (identity/presentation), and sexual orientation and race, ethnicity and racism.

Reporting on sex and gender	N/A
Reporting on race, ethnicity, or other socially relevant groupings	N/A
Population characteristics	N/A
Recruitment	N/A
Ethics oversight	N/A

Note that full information on the approval of the study protocol must also be provided in the manuscript.

Field-specific reporting

Please select the one below that is the best fit for your research. If you are not sure, read the appropriate sections before making your selection.

[x] Life sciences [ ] Behavioural & social sciences [ ] Ecological, evolutionary & environmental sciences

For a reference copy of the document with all sections, see nature.com/documents/nr-reporting-summary-flat.pdf

Life sciences study design

All studies must disclose on these points even when the disclosure is negative.

Sample size	A minimum of around 100 cells were analyzed by SMLM. Sample size is based on standards in the field (Bakshi, S., Siryaporn, A., Goulian, M. & Weisshaar, J. C. (2012) Mol Microbiol 85, 21-38); except for control experiments using chemostat growth conditions, in which a minimum of 30 cells were analyzed.
Data exclusions	Cell regions that identified more than one cell, superposed cells or incorrectly identified cell outlines were manually excluded from the SMLM analyses.
Replication	The minimum number of biological and technical replicates performed for each experiment is indicated in the text. As far as possible the same cultivation conditions and sample preparation was used in independent experiments yielding reproducible results.
Randomization	Not relevant to our study. Samples were not randomized. SMLM relies on large numbers and the biochemical measurements rely on quantitative measurements.
Blinding	Blinding is not relevant for our study. Cells for SMLM were chosen randomly by the investigator and analysed.

Reporting for specific materials, systems and methods

We require information from authors about some types of materials, experimental systems and methods used in many studies. Here, indicate whether each material, system or method listed is relevant to your study. If you are not sure if a list item applies to your research, read the appropriate section before selecting a response.

Materials & experimental systems

n/a	Involved in the study
☐	☒ Antibodies
☒	☐ Eukaryotic cell lines
☒	☐ Palaeontology and archaeology
☒	☐ Animals and other organisms
☒	☐ Clinical data
☒	☐ Dual use research of concern
☒	☐ Plants

Methods

n/a	Involved in the study
☒	☐ ChIP-seq
☒	☐ Flow cytometry
☒	☐ MRI-based neuroimaging

Antibodies

Antibodies used

anti-mCherry: Takara Bio Cat# 632543, RRID:AB_2307319; Takara Bio USA, Inc., Mountain View, CA, USA. Clone name not known.

anti-GFP: Antibodies-Online Cat# ABIN559689, RRID:AB_10852039 produced by Biomatik Corporation, Cambridge, Canada, Clone GF28R

anti-mouse IgG: Sigma-Aldrich Cat# A3562, RRID:AB_258091

Validation

Both antibodies specifically recognized the target fluorescent fusion proteins in western blot experiments. No signals were observed in the negative controls (cells lacking the corresponding protein), confirming the specificity of the antibodies used.

Validation available on the provider's website:

https://www.antibodies-online.com/antibody/559689/anti-Green+Fluorescent+Protein+GFP+antibody/

https://www.takarabio.com/products/antibodies-and-elisa/fluorescent-protein-antibodies/red-fluorescent-protein-antibodies, in the Image Data tab it is shown that the antibody detects PAmCherry on a mammalian cell lysate by western blot.
